# Geographical discrepancy in oral food challenge utilization based on Canadian billing data

**DOI:** 10.1186/s13223-022-00751-6

**Published:** 2023-01-17

**Authors:** Ala El Baba, Samira Jeimy, Lianne Soller, Harold Kim, Philippe Begin, Edmond S. Chan

**Affiliations:** 1 Division of Internal Medicine, Department of Medicine, Western University, 1151 Richmond St, London, ON N6A 3K7 Canada; 2 Division of Clinical Immunology and Allergy, Department of Medicine, Western University, London, ON Canada; 3 Division of Allergy and Immunology, Department of Pediatrics, University of British Columbia, Vancouver, BC Canada; 4 BC Children’s Hospital Research Institute, Vancouver, BC Canada; 5 Division of Clinical Immunology and Allergy, Department of Medicine, McMaster University, Hamilton, ON Canada; 6 Division of Allergy, Rheumatology and Immunology, Department of Pediatrics, CHU Sainte-Justine, Montréal, QC Canada; 7 Division of Allergy and Clinical Immunology, Department of Medicine, Centre Hospitalier de l’Université de Montréal, Montréal, QC Canada

**Keywords:** Oral food challenges, OFC, Food allergies, Canadian allergists, Barriers, Québec, Ontario, OHIP, RAMQ

## Abstract

**Background:**

Oral food challenges (OFC) confer the highest sensitivity and specificity in diagnosis; however, uptake has been variable across clinical settings. Numerous barriers were identified in literature from inadequate training to resource access. OFC utilization patterns using billing data have not been previously studied.

**Objective:**

The objective of this study is to explore the geographic differences in utilization of OFCs across Ontario and Québec using anonymized billing data from 2013 to 2017.

**Methods:**

Anonymized OFC billing data were obtained between 2013 and 2017 from Ontario Health Insurance Plan (OHIP) and Régie de l'Assurance Maladie du Québec (RAMQ). The number of OFCs was extracted by location, billings, and physician demographics for clinic and hospital-based challenges.

**Results:**

Over the period studied, the number of OFCs increased by 92% and 85% in Ontario clinics and Québec hospitals, respectively. For Ontario hospitals, the number of OFCs increased by 194%. While Québec performed exclusively hospital-based OFCs, after controlling for the population, the number of OFCs per 100,000 residents annually were similar to Ontario at 50 and 49 OFCs, respectively. The number of OFCs varied across the regions studied with an annual rate reaching up to 156 OFCs per 100,000 residents in urban regions and as low as 0.1 in regions furthest from city centers.

**Conclusion:**

OFC utilization has steadily increased over the last decade. There has been marked geographical discrepancies in OFC utilization which could be driven by the location of allergists and heterogeneity in their practices. More research is needed to identify barriers and propose solutions to them.

**Supplementary Information:**

The online version contains supplementary material available at 10.1186/s13223-022-00751-6.

## Background

The prevalence of food allergies (FA) is on the rise, affecting up to 9.3% of Canadians [1]. Suspected food allergies can lead to unnecessary food restrictions and anxiety surrounding accidental ingestions [2–4]. Prompt diagnosis of FA is critical as the mainstay of treatment is identifying and eliminating allergenic foods. Currently, the diagnosis of a FA typically consists of appropriate history, skin prick testing and serum immunoglobulin E levels to suspected allergens. While the objective tests confer a high sensitivity, they only have an estimated specificity of 60% which may generate false positives to food allergens being tested [4–6]. In contrast, oral food challenges (OFC) have a sensitivity and specificity approaching 100% [4, 7].

While OFCs are the gold standard for diagnosis, OFC uptake among practitioners has been variable. Grewie et al. surveyed 272 allergists in the USA, and determined that up to 40% of eligible patients were not offered OFC for early peanut introduction due to barriers such as lack of time (69.6%), lack of staffing support (51.8%), lack of space to conduct OFCs (46.3%), inexperience (16.5%) and concerns regarding hospital proximity (11.4%) [8]. Other factors that delayed OFC testing in eligible patients included concerns over process management, lack of comfort, poor reimbursement and inadequate training [9, 10]. Difference in physician compensation has also been found to affect OFC utilization [11]. From a patient perspective, OFCs have been deferred as patients were not interested in the food item (57%), patients feared they would develop a reaction (47%), or parents feared their child would have a reaction (31%) [12].

Finally, geographic barriers could also restrict access to OFCs. Generally, access to specialist care in Canada has been limited by their shortage as well as the distance between patients and their physicians. A recent paper published by Lee et al. advocated for the use of telemedicine to bridge Allergy and Immunology care to patients with limited access in remote communities [13].

Here, we hypothesize that the lack of access to OFCs is exacerbated by geography and corresponding deficit in Allergist coverage. We tested this hypothesis by exploring the discrepancy in the utilization of OFCs by geographical location as well as the trends in OFCs performed in community and hospital clinics using anonymized large-scale billing data from two provinces in Canada.

## Methods

This is a cross-sectional study that evaluated the change in OFC practice over recent years and differences between administrative regions. Billing data on OFCs conducted in Québec and Ontario between 2013 and 2017 was extracted from OHIP (Ontario Health Insurance Policy) and RAMQ (Régie de l'assurance maladie du Québec) databases which are the provincial health care systems for Ontario and Québec, respectively. For our study, 2017 was chosen as the end date for the extraction of OFC billing data since Oral Immunotherapy (OIT) was introduced after this time and visits were billed using OFC codes in Québec which would confound our outcomes. The data was anonymized, and a random half of the dataset was suppressed prior to extraction to ensure confidentiality [14].

For each OFC, we extracted the age and gender of the ordering physician, the age and gender of the patient, the region where the OFC was conducted, and the amount billed per OFC. OFC utilization was compared by year and by LHINs and Administrative Regions in Ontario and Québec, respectively. Government estimates of population for each LHINs and Administrative Regions in 2017 were used to calculate annual rate of OFCs per 100,000 residents and expressed on heat maps for each province. For Québec, the number of allergists with hospital positions were obtained for each administrative region through the province’s *Plan d’Effectif Médicaux*. and for Ontario, through the College of Physicians in Ontario (CPSO) between 2013 and 2017. Descriptive statistics were compiled for physician age, gender, and speciality, for patient age and gender as well as for the billing fees. The association between the number of OFCs and the number of allergists practising in an administrative region was assessed with linear regression.

## Results

In Ontario, 33,788 OFCs were performed during the study period. There were 24,423 OFCs (72.24%) performed in community clinics and 9384 (27.76%) performed in hospital (Additional file 1: Table S1). Hospital OFCs were generally performed by younger physicians (average 41.0 ± 8.0 y) than those performed in community (47.2 ± 10.4y) (p < 0.00001). They were also more frequently performed by female physicians (42%) compared to those performed in community setting (34%) (OR = 1.43, p < 0.0005).

**Fig. 1:**
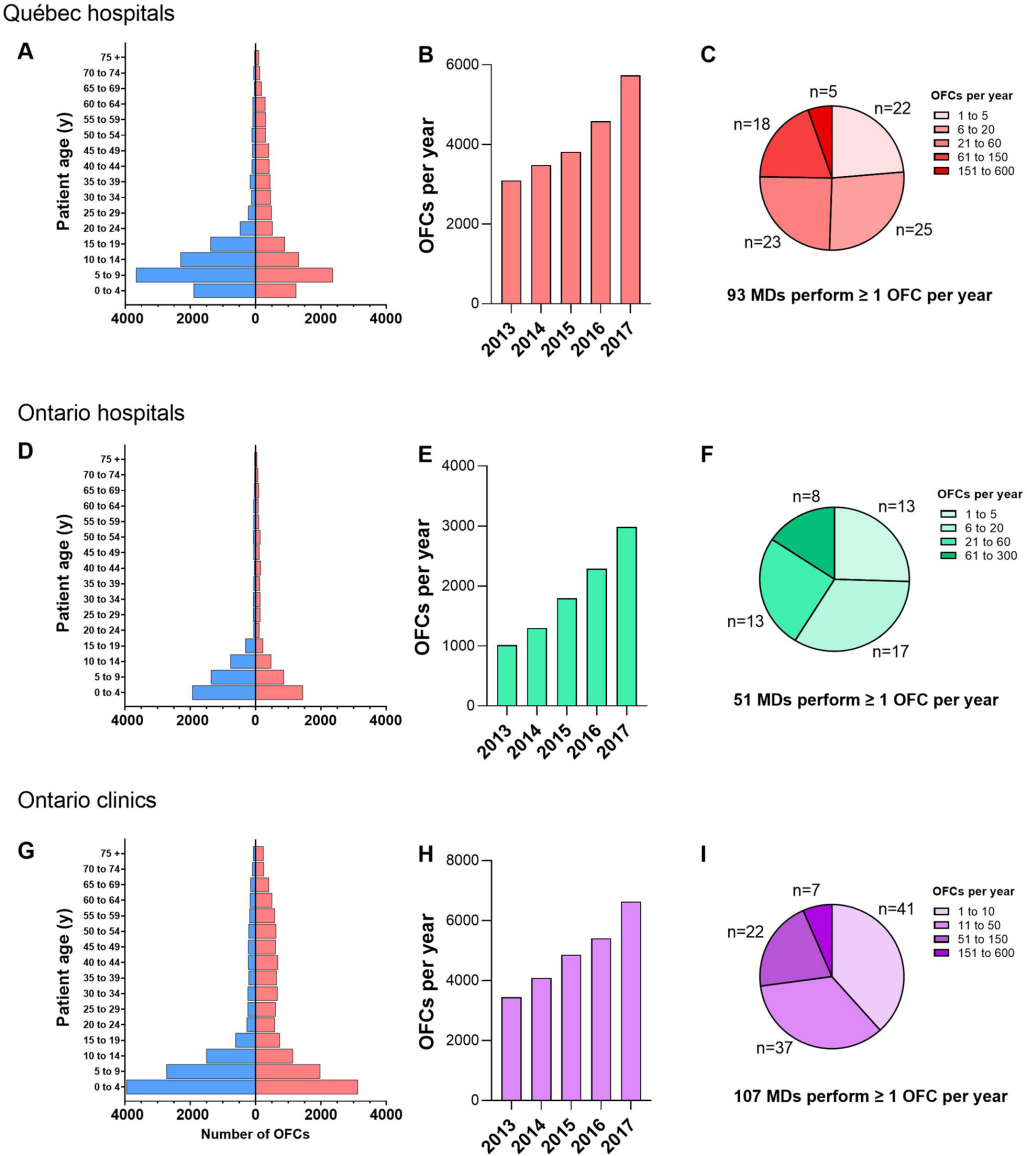
Oral Food Challenges in Québec and Ontario between 2013 and 2017. **A**, **D** and **G** present the number of oral food challenges (OFCs) performed in male (blue) vs female patients (red) according to age groups, in Québec hospitals, Ontario hospitals and Ontario clinics during the study period. **B**, **E** and **H** indicate the number of OFC performed for each year. **C**, **F** and **I** present the average number of OFCs performed per year by physicians in the two provinces. Each portion of the pie charts indicates the proportion of physicians with the various productivity. Physicians who performed an average of less than 1 OFC per year were excluded

In Québec, 20,716 OFCs were billed during the study period, all of which were performed in hospital where currently there is no billing code available for office-based challenges. Physician demographics were not available. About 51% of OFCs were billed under the hybrid remuneration plan involving a baseline salary and partial fee-for-service whereas the rest were billed using traditional fee-for-service plan. For Québec hospitals, 61% of OFCs were performed in children younger than 15 years old, compared to 72% in Ontario hospitals and 59% in Ontario community clinics. In the pediatric population, 58%, 59% and 57% were performed in male patients in Québec hospitals, Ontario hospitals and Ontario clinics, respectively, compared to 38%, 38% and 29% in the adult population (Fig. 1). In both provinces, there was a steady increase in the number of OFCs performed annually (Fig. 1). Over the 5-year period studied, the number of OFCs increased by 92% and 85% in Ontario clinics and Québec hospitals, respectively. Whereas for Ontario hospitals, the number of OFCs almost tripled at a 194% increase. In both Québec and Ontario, there was large variability in the total number of OFCs performed by each physician individually, with the majority of OFCs being performed by less than a quarter of allergists (Fig. 1).

**Fig. 2:**
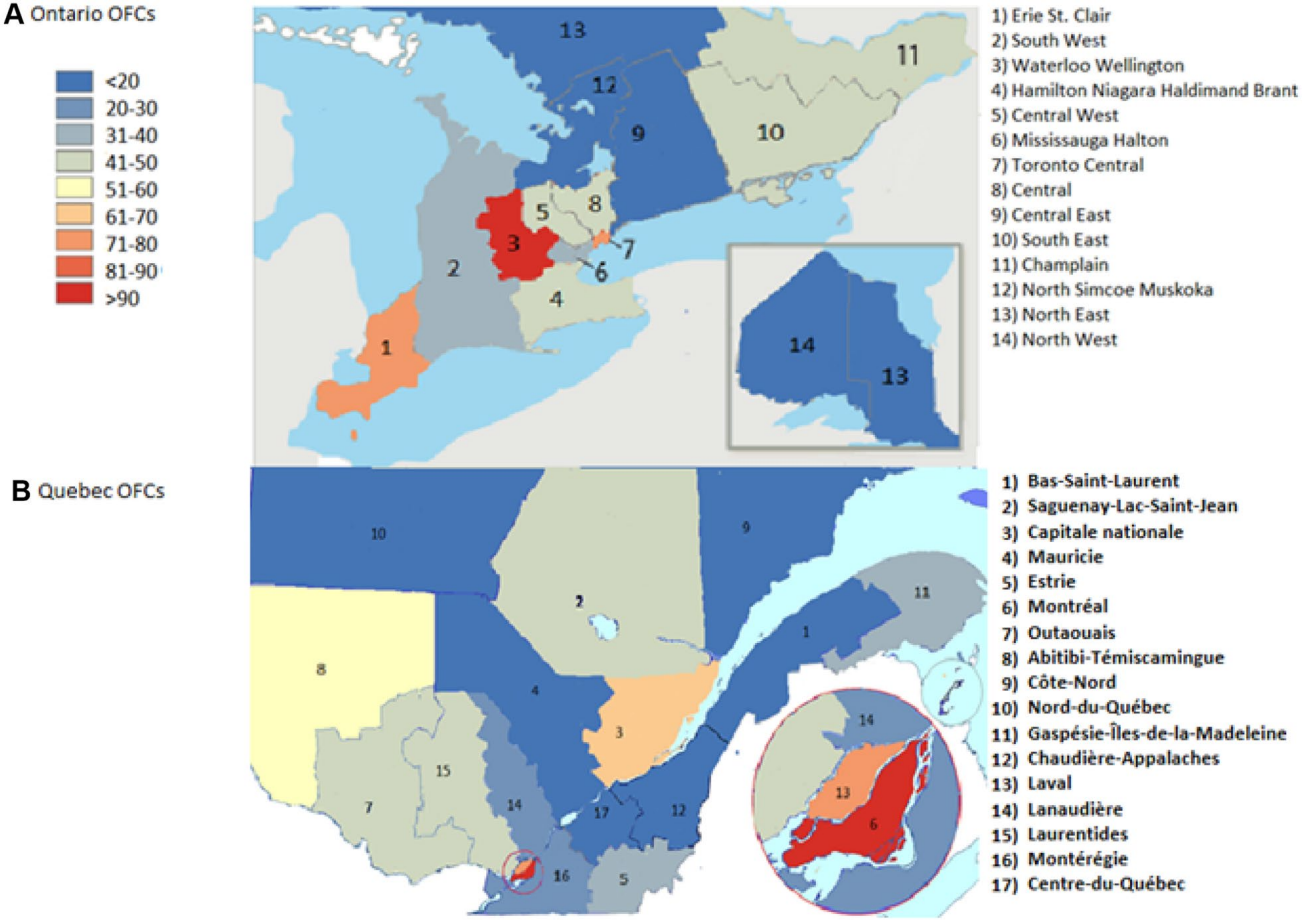
Heat Map demonstrating number of total OFCs conducted annually per 100,000 residents in Ontario and Québec

In Ontario, approximately 50% of all OFCs were performed in 3 LHINs: Waterloo Wellington at 17.89%, Toronto Central at 17.09% and Central at 11.49%, respectively. However, the most densely populated LHINs were Hamilton Niagara, Central East and Champlain. In Québec, the majority of OFCs were performed in Montréal totalling 52.95% of the province’s OFCs (Fig 2, Tables 1 and 2).

**Table 1:** Number of OFCs performed in Ontario and corresponding number of allergists and population by LHIN between 2013 and 2017

LHIN	N of OFCs	%	Total population	Number of allergists
Erie St-Clair	2746	8.12	627,633	4
South West	1866	5.52	953,261	5
Waterloo Wellington	6051	17.89	766,109	6
Hamilton Niagara	3388	10.02	1,399,073	19
Central West	2000	5.91	922,255	3
Mississauga	1973	5.83	1,164,740	12
Toronto central	5780	17.09	1,232,258	18
Central	3886	11.49	922,255	18
Central East	1121	3.31	1,550,531	10
South East	1007	2.98	482,391	5
Champlain	3268	9.66	1,292,639	10
North Simcoe Muskoka	421	1.24	464,406	4
North East	12	0.04	551,801	0
North West	306	0.90	228,339	1
Total	33,825	100	13,448,494	115

**Table 2:** Number of OFCs performed in Québec Hospitals and corresponding number of allergists and population by administrative region between 2013 and 2017

LHIN	N of OFCs	%	Total population	Number of allergists
Bas-Saint-Laurent	138	0.67	197,385	0
Saguenay-Lac-Saint-Jean	602	2.91	276,368	0
Capitale-Nationale	2576	12.43	729,997	10
Mauricie et Centre-du-Québec	60	0.29	242,399	0
Estrie	614	2.96	319,004	4
Montréal	10,970	52.95	4,098,927	37
Outaouais	788	3.80	382,604	2
Abitibi-Témiscamingue	412	1.99	146,717	1
Côte-Nord	0	0.00	92,518	0
Nord-du-Québec	0	0.00	44,561	0
Gaspésie-Îles-de-la-Madeleine	142	0.69	90,311	0
Chaudière-Appalaches	188	0.91	420,082	1
Laval	1502	7.25	437,413	3
Lanaudière	676	3.26	494,796	3
Laurentides	172	0.83	589,400	2
Montérégie	1876	9.06	1,507,070	7
Nunavik	0	0.00	13,188	0
Terres-Cries-de-la-Baie-James	0	0.00	17,141	0
Total	20,716	100.0	8,164,361	70

**Fig. 3:**
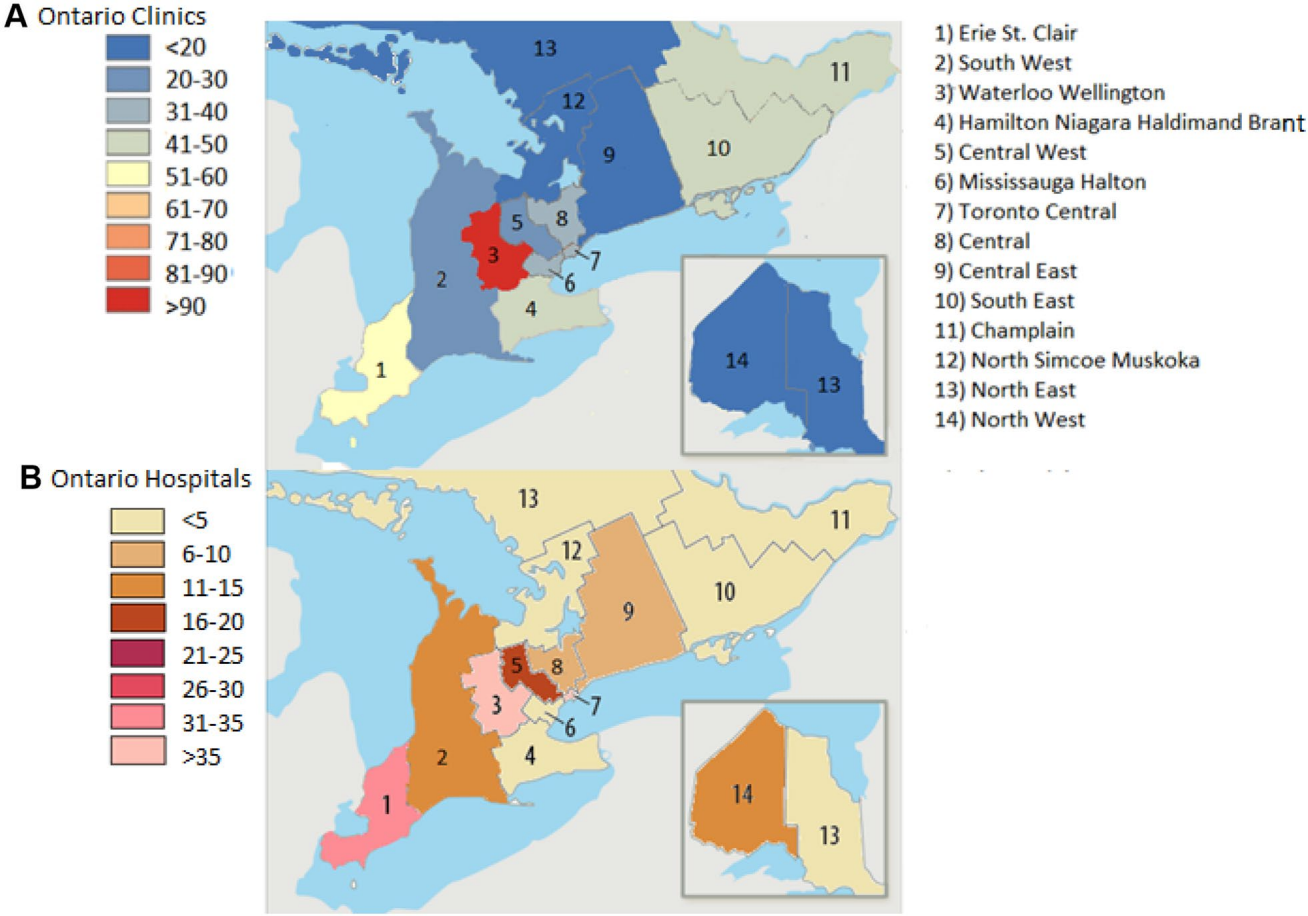
Heat Map demonstrating number of clinic and hospital-based OFCs conducted annually per 100,000 residents in Ontario

**Fig. 4:**
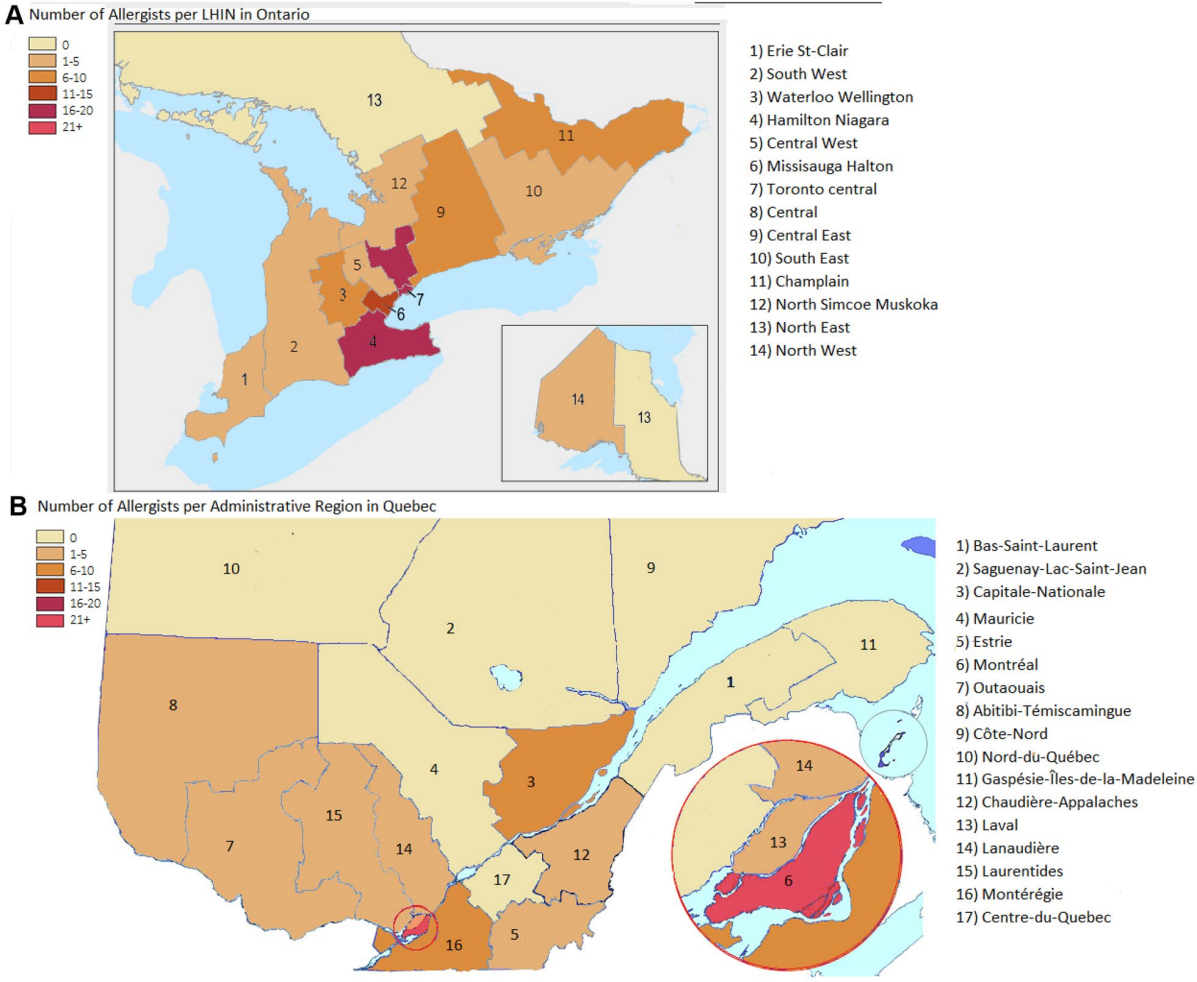
Heat map demonstrating the number of Allergists practicing per LHIN in Ontario and per Administrative Region in Québec

The geographical difference was even more striking when considering the rate of OFCs performed per 100,000 residents, annually (Additional file 1: Table S2 and S3). In Ontario, Waterloo-Wellington LHIN had a rate of 156 OFCs per 100,000 residents per year—well above Toronto Central and Erie St. Clair’s annual rates at 89 and 84 OFCs per 100,000 residents, respectively (Fig. 3). When looking at the number of practicing allergists per LHIN, Hamilton Niagara, Toronto Central and Central LHINs had the most allergists at 19, 18, 18 allergists each, respectively (Fig. 4). Across Ontario, the average number of OFCs conducted annually per 100,000 residents was 49 challenges. Similarly, the average number of OFCs performed annually per 100,000 residents in Québec across all the administrative regions was 50, with 110 OFCs performed annually per 100,000 residents in Montreal, followed by 70.6 and 69.9 OFCs in Laval and Capitale-Nationale, respectively (Fig. 3). When looking at the number of Allergists per Administrative Region in Québec–Montreal, Capitale-Nationale and Montérégie had the most allergists at 37, 10 and 7 each, respectively (Fig. 4).

**Fig. 5:**
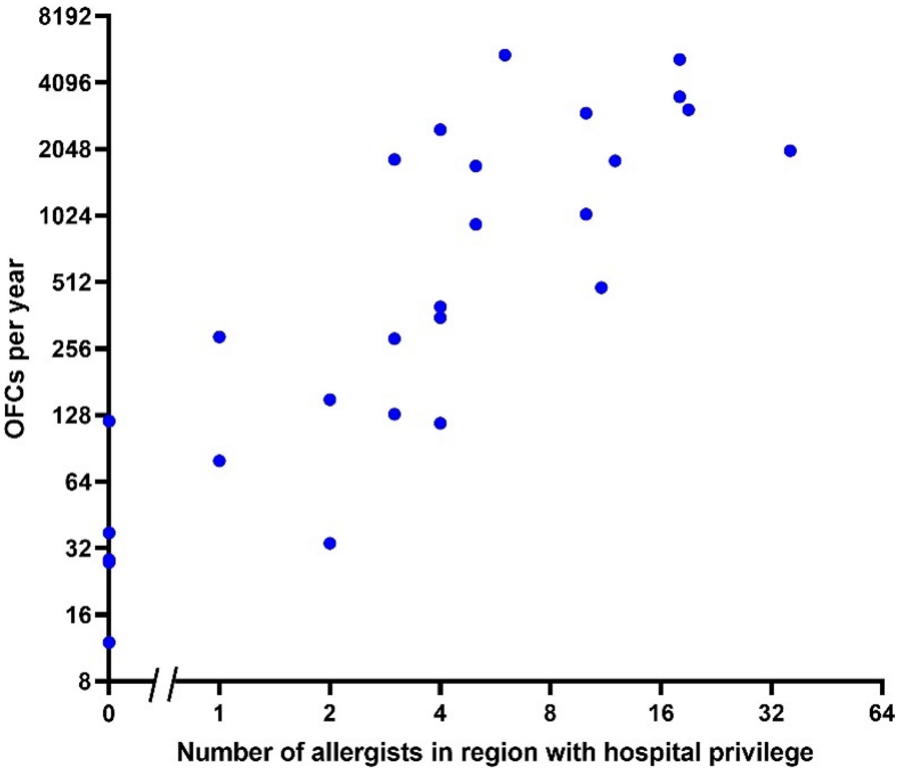
Correlation between number of allergists with hospital privileges and number of OFC performed in an administrative region for the provinces of Québec and Ontario

For both Québec and Ontario, the rate of OFCs was strongly correlated with the number of allergists having hospital privileges in the corresponding administrative regions and LHINs (R^2^ = 0.98). There were a few administrative regions with no allergists who had OFCs performed by specialists who had hospital privileges such as pediatricians (Fig. 5).

## Discussion

Using annual OFC billing data for Québec and Ontario from 2013 to 2017, we observed a steady increase in the uptake of OFCs within both provinces. However, we also found a significant discrepancy in OFC utilization when the data was analyzed by geographic region. This finding supports the Canadian Society of Allergy and Clinical Immunology statement made in the context of OIT guideline formation which describes “a situation of low capacity and disparity in access to care for the accurate diagnosis and proper management of food allergy” [15]. To the best of our knowledge, this is the first time that this disparity in access to allergy care is demonstrated in a quantitative manner using anonymized billing data, which raises significant concerns in terms of equity.

The heterogeneous distribution of allergists across the regions studied cannot explain the geographical disparities by itself. In fact, we found that the number of OFCs performed per year could vary by a 100-fold depending on the allergist. In Ontario, the highest absolute number of OFCs were performed in a Waterloo Wellington, despite having a smaller population and lower number of practicing allergists compared to its neighboring, densely populated LHINs such as Toronto Central and Mississauga Halton [16].

This disparity in allergists’ practice with regards to OFCs has a crucial implication in terms of macroscopic planning. The provincial governments’ strategy to promote access in rural areas has been to restrict practice of newly certified allergists to regions that lack allergists. While this is clearly an important factor, we show here that having one or many allergists does not guarantee that the clinical offer in OFCs will necessarily follow. Qualitative studies can provide important clues as to why some allergists are reluctant to offer OFCs. This could reflect ongoing issues with resource access, perception of risk and reimbursement concerns which were all cited by American physicians as the top 3 barriers limiting their performance of office-based OFCs [10]. These barriers were echoed in a Canadian study surveying allergists in British Columbia where 72.6% of physicians performing OFCs reported lack of resources such as office space and support staff as influential barriers limiting OFC performance. Equally, 72.6% reported that the creation of standard guidelines for hospital versus community OFCs would influence them to perform more OFCs [17].

Interestingly, training experience has commonly been cited as a barrier to OFC performance where inadequate exposure to OFCs in fellowship limited OFC performance in clinical practice [4, 9]. One of the earliest studies investigating OFC barriers and uptake was published by Pongracic et al. in 2009 which showed that 45% of responding American Allergists reported never having personally performed OFCs during fellowship training [10]. A follow-up to this study conducted by Grewie et al. in 2020 showed that in comparison to the 2009 study by Pongracic et al. significantly more providers who performed OFCs in fellowship, offered OFCs in clinical practice [18].

Allergen challenges are procedures typically restricted to Allergy and Immunology subspecialists where the skill is taught exclusively within Clinical Immunology and Allergy specialty training programs. However, given the high prevalence of food and drug allergies and the limited number of allergists, it is currently difficult for allergists to manage all cases themselves. New interdisciplinary models need to be explored to cater to the needs of patients with food allergies. For example, the administrative region with the third highest rate of OFCs in Québec was Saguenay-Lac-St-Jean (03) despite having no practicing allergists. It was also an administrative region where OIT was successfully implemented once it was available in the province. This was made possible by pediatricians who preformed OFCs with direct access to an allergist in Montréal who would provide clinical direction and discuss challenging cases based on up-to-date clinical practices so that patients receive specialized care despite the geographical barriers that limit their access to an allergy subspecialist locally.

The overall increase in OFC uptake likely reflects an increase in their demand following the recent shift in practice towards a greater focus on formal diagnosis of food allergies, support to early introduction of food allergens and threshold determination prior to the initiation of oral immunotherapy [18, 19]. While technically there are two clinical settings to conducting OFCs, the restriction of Québec OFCs to hospitals ultimately did not limit the overall number of challenges completed as the number of OFCs per 100,000 residents were similar in Ontario and Québec. When comparing hospital and community based OFCs in Ontario, allergists performing OFCs in hospital were found to be younger and more frequently females, which may be representative of the new generation of allergists. One limitation here is that we cannot compare the type of challenges performed in the various settings based on allergy severity and perceived risk.

A little over a half of OFCs in Québec were billed using the hybrid remuneration plan. It is likely a reflection that this plan is used in academic centers, which perform most of the OFCs in the province. Contrary to academic centers, community hospitals often only allocate a limited number of days per month for allergists to perform challenges and they may not have access to dedicated full-time nurses with the experience required to perform a large amount of OFCs simultaneously.

Limitations of our study include the generalizability of our findings as the data we collected was restricted to the provinces of Ontario and Québec. While all Canadian provinces have public healthcare models funded by each of their respective provincial governments, there could be regional variations in the rate of food allergies, patient demographics, physician demographics and access to allergists that could affect OFC utilization patterns. Moreover, billing codes for OFC’s could vary considerably in different provinces [20]. Given the discrepancy in OFC utilization we reported, future considerations include the introduction of pilot projects that aim to improve access to OFCs in LHINs and Administrative Regions with limited resources to perform OFCs that meet the needs of their communities. In the era of telemedicine, allergists can utilize virtual visits to supervise low risk OFCs for patients who have an epinephrine device at home and who understand the signs and symptoms of a reaction and when to treat [21]. Such initiatives could also be expanded to virtually connect community pediatricians or internists to allergists from academic centers to direct higher risk in-office OFCs in order to increase their utilization and improve equity in management of food allergies, which are on the rise (Additional file 1).

## Conclusion

In conclusion, while we observed a steady increase in the performance of OFCs across Ontario and Québec, there were major discrepancies in their clinical offer across the regions studied, irrespective of the practice setting. Addressing the various barriers to OFCs is essential, but likely insufficient on its own to ensure fair access to allergy care. The responsibility of performing OFCs will likely need to be shared in part with other practitioners in areas of limited allergist access, where allergists play a role in overseeing the transition and supporting the practice.

## Supplementary Information

**Additional file 1: Supplementary Tables:** **Table S1.** Demographics of physicians performing OFCs in Ontario community and hospital clinics between 2013 and 2017. **Table S2.** Number of OFCs preformed annually per 100,000 residents in Ontario Clinics and hospitals across the LHINs. **Table S3.** Number of OFCs preformed annually per 100,000 residents in Quebec hospitals across the LHINs.

## Data Availability

The data that support the findings of this study are available from OHIP and RAMQ. Restrictions apply to the availability of these data, which were used under license for the current study, and so are not publicly available.

## Abbreviations

OFC: Oral food challenge
FA: Food allergies
OHIP: Ontario Health Insurance Plan
RAMQ: Régie de L'assurance Maladie du Québec
AAAAI: American Academy of Allergy, Asthma, and Immunology
CSACI: Canadian Society Allergists and Clinical Immunologists
LHIN: Local Health Integration Network
OIT: Oral immunotherapy
