# Supplementary material for: Geographical discrepancy in oral food challenge utilization based on Canadian billing data
Source: Allergy Asthma Clin Immunol. 2023 Jan 17;19:5. doi: 10.1186/s13223-022-00751-6 (PMC9843933; doi:10.1186/s13223-022-00751-6)
Supplement: Supplementary file 1 — Additional file 1: Supplementary Tables: Table S1. Demographics of physicians performing OFCs in Ontario community and hospital clinics between 2013 and 2017. Table S2. Number of OFCs preformed annually per 100,000 residents in Ontario Clinics and hospitals across the LHINs. Table S3. Number of OFCs preformed annually per 100,000 residents in Quebec hospitals across the LHINs. [file 13223_2022_751_MOESM1_ESM.docx]

**Additional file:**

**Table S1 -** Demographics of physicians performing OFCs in Ontario community and hospital clinics between 2013-2017

| **Physician Age** |  | **Clinics** | **Hospitals** |
| --- | --- | --- | --- |
|  | **Average (+/- SD)** | 47.20 +/- 10.42 | 41.00 +/- 8.00 |
|  | **Median** | 46.00 | 41.00 |
|  | **Range** | 29.00 – 81.00 | 29.00 – 80.00 |
|  | **Male**  **Average (+/- SD)**  **Median**  **Range** | 49.42 +/- 24.95  49.00  31.00 – 81.00 | 44.11 +/- 22.82  42.00  31.00 - 80.00 |
|  | **Female**  **Average (+/-SD)**  **Median**  **Range** | 42.79 +/- 20.77  41.00  29.00 – 73.00 | 41.54 +/- 20.92  40.00  29.00 - 71.00 |
| **Total OFC between 2013-2017** |  |  |  |
|  | **N (%)** | 15137 (61.98%) | 9286 (38.02%) |

**Table S2** - Number of OFCs preformed annually per 100,000 residents in Ontario Clinics and hospitals across the LHINs

| **LHIN** | **OFC per year per 100,000 residents** | **Clinic OFCs** | **Hospital OFCs** |
| --- | --- | --- | --- |
| Erie St-Clair | 84.4 | 55.3 | 29.2 |
| South West | 39.2 | 28.2 | 11.0 |
| Waterloo Wellington | 156.1 | 112.2 | 43.9 |
| Hamilton Niagara | 47.3 | 42.3 | 4.9 |
| Central West | 42.0 | 25.5 | 16.5 |
| Mississauga | 31.7 | 31.6 | 0.1 |
| Toronto central | 88.9 | 36.1 | 52.8 |
| Central | 41.5 | 33.1 | 8.4 |
| Central East | 14.2 | 8.8 | 5.4 |
| South East | 40.5 | 40.2 | 0.3 |
| Champlain | 48.9 | 46.8 | 2.1 |
| North Simcoe Muskoka | 18.1 | 17.8 | 0.3 |
| North East | 0.2 | 0.0 | 0.2 |
| North West | 25.8 | 2.6 | 23.2 |
| **Ontario Average** | **48.8** | **35.2** | **13.6** |

**Table S3** - Number of OFCs preformed annually per 100,000 residents in Quebec hospitals across the LHINs

| **Région administrative** | **OFC per year per 100,000** |
| --- | --- |
| Bas-Saint-Laurent | 13.8 |
| Saguenay-Lac-Saint-Jean | 43.4 |
| Capitale-Nationale | 69.9 |
| Mauricie et Centre-du-Québec | 2.4 |
| Estrie | 38.1 |
| Montréal | 109.7 |
| Outaouais | 40.9 |
| Abitibi-Témiscamingue | 55.8 |
| Côte-Nord | 0.0 |
| Nord-du-Québec | 0.0 |
| Gaspésie-Îles-de-la-Madeleine | 30.9 |
| Chaudière-Appalaches | 8.9 |
| Laval | 70.6 |
| Lanaudière | 27.3 |
| Laurentides | 5.8 |
| Montérégie | 24.7 |
| **QUÉBEC** | **50.1** |
